# The Role of Extracellular Vesicles in Neuropsychiatric Disorders

**DOI:** 10.34172/apb.025.46020

**Published:** 2025-10-11

**Authors:** Behnaz Mirzaahmadi, Reza Rahbarghazi, Mohammad Karimipour

**Affiliations:** ^1^Student Research Committee, Tabriz University of Medical Sciences, Tabriz, Iran; ^2^Department of Applied Cell Sciences, Faculty of Advanced Medical Sciences, Tabriz University of Medical Sciences, Tabriz, Iran; ^3^Department of Anatomical Sciences, Tabriz University of Medical Sciences, Tabriz, Iran

**Keywords:** Extracellular vesicles, Neuropsychiatric disorders, Therapeutic effects

## Abstract

**Purpose:**

There are effective treatments available for neuropsychiatric disorders; however, numerous factors such as misdiagnosis, delayed diagnosis, varying types of onsets and progression of disorder, as well as long timeframes between diagnosis and treatment, all serve to limit the ability of these treatments to reduce symptoms. This article discusses the unique theragnostic aspects of extracellular vesicles (EVs) in relation to these clinical considerations.

**Methods:**

An extensive review of the literature was conducted to examine recent findings about EVs and their potential role in early diagnosis and targeted treatment of neuropsychiatric disorders, to examine the biological aspects, diagnostic and therapeutic capacity.

**Results:**

Pathological states radically change the composition of EVs and their cargo delivery, thereby providing a dynamic disease state. EVs can cross biological barriers, have systemic distribution, provide low immunogenicity and toxicity, and all serve to represent the beginnings of identifying early biomarkers and future methods for delivering therapy needed to administer neuroactive drugs/delivery methods.

**Conclusion:**

In summary, EVs have potential as a theragnostic platform for neuropsychiatric diseases. Because they offer passive, non-invasive, and actionable diagnostic insights, EVs can be applied to therapeutically manage neuropsychiatric disorders, which could provide a solution to issues faced in clinical practice, such as late-stage diagnosis and poor treatment compliance. To fully understand the prospect of EVs in neuropsychiatric practice, more translational research and clinical validation are required.

## Introduction

 Neuropsychiatric disorders include a range of complex and debilitating conditions that significantly impact patients’ lives with heavy socioeconomic burdens.^1^ Among neuropsychiatric disorders, major depressive disorder (MDD), schizophrenia (SCZ), bipolar disorder (BD), and Alzheimer’s disease (AD) are the most common in the human population. Still, their etiology remains poorly understood due to ethical issues, practical challenges, the complex biology of higher brain function, and the weak validation of animal models.^2^ Neuroinflammation, oxidative stress, neurodegeneration, and the modulation of specific signaling pathways and neurotransmitters are the primary causes of the onset and progression of these disorders.^3,4^ Currently, clinical manifestations, symptoms, and cognitive tests are still used to diagnose individuals suspected of having the condition.^5^ Despite recent progress, the absence of accurate objective signs or biomarkers has led to the possibility of misdiagnosis and delays in initiating treatment protocols.^6^ To develop effective therapeutic protocols, understanding the underlying cellular mechanisms of neuropsychiatric disorders is crucial. Consequently, ongoing studies have focused on identifying new targets for drug development within the central nervous system (CNS).

**Figure 1:**
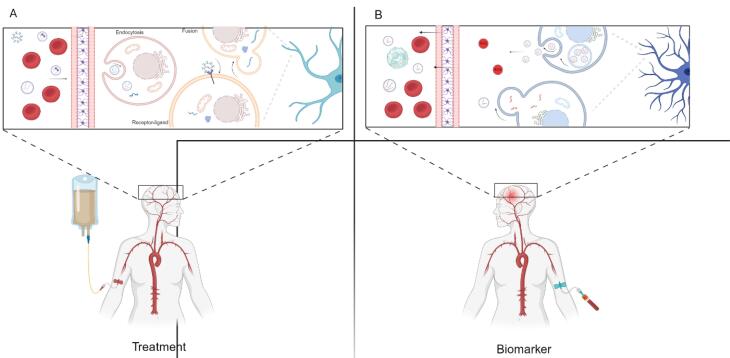
EVs have essential roles as biomarkers and in treatment. A. EVs hold promise as therapeutic agents for neuropsychiatric disorders, offering targeted delivery of therapeutic cargo to affected brain regions. B. EVs offer diagnostic potential, serving as biomarkers for early detection and monitoring of neuropsychiatric disorders

 The ability of extracellular vesicles (EVs) to provide important diagnostic highlights or markers, while simultaneously delivering the therapeutic agent directly to the targeted site, is a significant benefit for the patient, allowing for the integration of both diagnostic and potential treatment delivery interventions in a targeted manner. The use of this system would also provide physicians with real-time evidence of the intervention’s effectiveness, enabling them to make adjustments based on patient-specific needs.^7^ This review will examine both aspects of EV function by providing a summary of key biomarkers with diagnostic potential in neuropsychiatric disorders and discussing the potential adoption of EVs as therapeutic agents in modulating disease processes (Figure 1). Importantly, we will consider the integration of these important and innovative diagnostics and therapeutic functions to be a proper theragnostic within clinical neuropsychiatry, despite conflicting evidence.

## EVs in CNS

 EVs are an emerging field of biological research with the potential for treating neuropsychiatric disorders.^8^ EVs are endosomal, small, membrane-bound structures released by almost every cell into the extracellular matrix (ECM). EVs, especially exosomes (Exos), have garnered considerable attention due to their ability to transport signaling molecules, including proteins, lipids, and nucleic acids, between cells.^9^ These vesicles have been shown to play roles in neuroplasticity, neuroprotection, and improvement of neural dysfunction.^10^ Emerging data suggest that EVs have substantial roles in the pathophysiology and physiology of neuropsychiatric disorders.^11^ Within the CNS, EVs are produced by various cell types, including glial cells and neurons.^12^ For instance, as part of neuroimmune interactions, inflammatory astrocytes can produce EVs that can damage neurons.^13^ Of note, these particles can cross the biological barriers, such as the blood-brain barrier (BBB). It was suggested that the juxtaposition of EVs with endothelial cells (ECs) leads to the activation of several subcellular mechanisms, such as endocytosis, micropinocytosis, phagocytosis, and direct plasma fusion, resulting in EVs crossing the BBB.^14^ It should not be forgotten that their cargoes may also affect how and by which mechanisms EVs cross the BBB.^11^ EVs are also involved in the removal of waste byproducts in the CNS, helping the host cells eliminate aggregates of neurotoxic substances and reducing drug resistance by bypassing the P-glycoprotein drug efflux pathway.^15,16^ Besides, EVs play crucial roles in neurodevelopment and the homeostasis of the brain’s neuronal function and secretion.^17^ The uptake of EVs by target neuronal cells is dependent on lipid components in their membranes, such as sphingomyelin and phosphatidylserine.^18^

**Figure 2:**
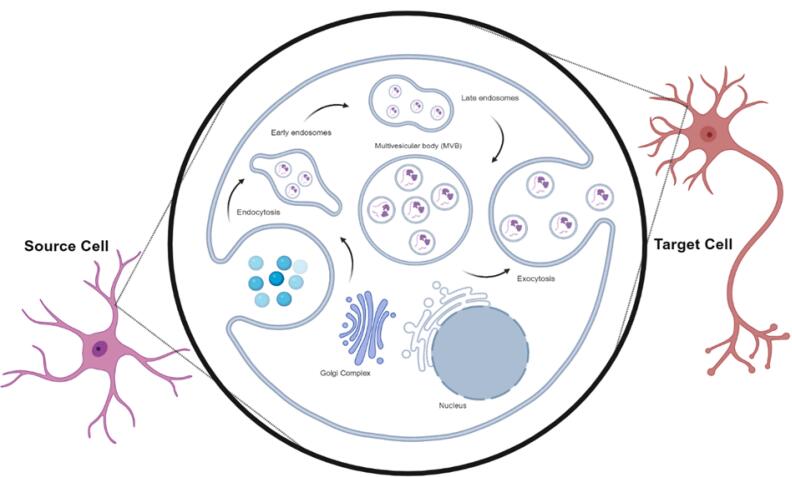
Exosome biogenesis. The internalized Exos are directed into early endosomes induced through cell membrane invagination. In the following steps, the invagination of early, late endosomes, and multivesicular bodies (MVBs) leads to the generation of several intraluminal vesicles (ILVs). The fusion of MVBs with the cell membrane contributes to the release of ILVs into the ECM, which are subsequently referred to as Exos, belonging to EVs

 EV biogenesis encompasses multiple molecular pathways that orchestrate the uptake of EVs, their fusion with endosomal systems, and the release of their cargo into the cytosol (Figure 2).^19^ In this process, EV ligands can bind to receptors on the recipient cells, activating entry mechanisms such as endocytosis, phagocytosis, and micropinocytosis.^20^ Following arrival in the cytosol, EV genomics, such as RNA, can alter host cell functions by changing gene expression or affecting certain signaling pathways.^21^ Among various genomic cargoes, EV miRNAs are valid biomarkers for detecting neuropsychiatric disorders.^22^ Interestingly, nearly 70% of body miRNAs are produced in the brain.^23^ These features demonstrate the theragnostic importance of miRNAs in the pathophysiology of several CNS pathological conditions. Other genetic elements, lncRNAs and circRNAs, also exist inside the EVs.^24^ LncRNAs with an average size of more than 200 nucleotides can regulate transcription, metabolism, signaling pathways, protein function, and RNA stability.^25^ circRNAs, covalently closed RNAs, function as protein scaffolds or miRNA sponges to regulate several cell functions.^26^ Among the various RNA types, circular RNAs (circRNAs) are more resistant to enzymatic degradation and thus possess longer lifespans, making them more reliable biomarkers for chronic pathologies and therapeutic targets.^27^

 EV biomarkers provide essential data from the parent cells. For instance, the existence of glutamate-aspartate transporters indicates that these EVs originate from glial cells.^28^ In the CNS, L1CAM is used to discriminate neuron EVs from other cell sources.^29^ In addition to their diagnostic potential, EVs can be modified and bioengineered to transfer signaling biomolecules and therapeutics, thereby influencing gene expression, translation, and signaling pathways for the treatment of specific pathological conditions.^30^ Specific physicochemical properties, such as a slight negative zeta potential, flexible cytoskeleton, and low immunogenicity, support their application as nanocarriers for drug delivery to the CNS.^31^ These properties indicate their superiority compared to synthetic nanocarriers that are cytotoxic or cleared by the reticuloendothelial system.^32^

 For sampling, EVs can be obtained from different biofluids for various scientific purposes. Using liquid biopsy, EVs have been obtained from the postmortem brain, cerebrospinal fluid (CSF), and blood. To enrich these particles, techniques such as size exclusion chromatography, immunoisolation, differential ultracentrifugation (UC) steps, microfiltration, and microfluidic systems are available.^33^ However, the isolation methods can, in part but not completely, alter the structural integrity and molecular contents, and thus, there is an urgent demand to develop standardized extraction protocols to minimize unintended *in vitro* changes. The process used to isolate the EVs will also impact, to some degree, the sensitivity and specificity of the assay.^34^ UC is the standardized method, which is great in its specificity and lower in contamination, but also yields less.^35^ Polyethylene glycol (PEG) is a precipitation-based technique that is quicker and yields more proteins/particles, but typically co-isolates more proteins/particles, thereby lowering specificity.^36^ Size exclusion chromatography (SEC) balances yield with low contamination and is less damaging to vesicle structure, making this method more ideal for studies that attempt to detect sensitive biomarkers.^37^ Antibody-coated magnetic beads, combined with immunoaffinity-based methods, can increase the specificity and sensitivity of EV subtype separation by explicitly utilizing the surface markers of EVs; however, this approach often requires some prior knowledge of EV surface markers.^38^ Studies that use magnetic beads are limited by the volume of the samples, which can potentially contaminate the enriched EVs compared to other methods.^37^ Preanalytical variables, including biofluid (e.g., plasma, serum, cerebrospinal fluid), storage temperature, freeze-thaw cycles, and handling, will impact the integrity of EVs, thereby affecting the reliability of biomarkers and often leading to inconsistencies in results. In the examples, improper sample storage can alter or aggregate EVs, thus limiting the potential for meaningful detection and any molecular profiling or experimental use of EVs thereafter.^39^ If nothing else, standardizing the steps involved in EV isolation protocols and preanalytical factors can only make biomarker studies more informative.

## Role of EVs in SCZ

 SCZ, a severe neuropsychiatric disorder with a prevalence of about 1% of the human population, exhibits a high recurrence and disability rate.^40^ A major predictor of disability and functioning in SCZ is cognitive deficits.^41^ Globally, the life expectancy in SCZ patients ranges between 10 and 20 years, and for some patients leads to suicide.^42^ It has been thought that a close interaction between genetic elements and the environment underpins these conditions.^4^ SCZ patients are still diagnosed between adolescence and early adulthood by clinical interviews and observations, with a risk of delay and misdiagnosis when starting treatment.^40^ Timely diagnosis is an essential step toward recovery. Medications using antipsychotics mainly alleviate symptoms such as hallucinations, but don’t have a remarkable impact on cognitive symptoms such as learning and attention.^4^ Molecular investigations have revealed that inflammatory response, neurotrophic deficit, and epigenetic regulation have been implicated as potential biomarkers of SCZ.^43^ Several candidate molecules have been proposed as SCZ biomarkers using “omics” methods, including proteomics, transcriptomics, and metabolomics.^44^ Despite the existence of several proposed biomarkers for SCZ, the diagnosis of SCZ based on candidate biomarkers remains elusive. Therefore, it is crucial to understand the etiology of this disease in order to develop novel biomarkers that aid in diagnosis and prognosis. Emerging data have indicated the presence of valid signaling molecules, such as miRNAs, circRNAs, metabolites, and proteins, inside the EVs under SCZ conditions.^45,46^ The differential expression of 44 circRNAs involved in stress response and pathogenesis has been verified in the plasma of SCZ patients and healthy counterparts. It is believed that EV miRNAs act as epigenetic regulators during the onset of SCZ.^47^

 SCZ patients had significantly higher levels of 11 miRNAs compared to healthy controls, as determined by genome-wide interactive analysis of blood exosome miRNA data. Among these miRNAs, hsa-miR-206 exhibited the highest levels, and animal models demonstrated that hsa-miR-206 has detrimental effects on cognitive functions by disrupting brain-derived neurotrophic factor (BDNF) mRNA and protein expression. As a result, the abnormalities in the brains of SCZ patients are associated with disturbances of neurotrophic factors.^48^ There is evidence that SCZ has a linkage with excessive dopaminergic activity in the mesolimbic tract, gamma-aminobutyric acid (GABA)-ergic inhibitory activities, decreased dopamine in the prefrontal cortex, and reduction of mesocortical dopaminergic neurons.^49^ In a study conducted by Funahashi et al, microarray analysis revealed 18 down-regulated and 13 up-regulated miRNAs in SCZ conditions linked to brain development. Of note, the non-treatment-resistant SCZ group exhibits a significant reduction in miR-675-3p expression.^50^ But this study has limitations that included a small sample size for the microarray cohort (there were only 9 patients and 9 controls), and the SH-SY5Y cell line used for clozapine stimulation may not entirely recapitulate the complex environment in vivo within the brain, including cell-specific effects.

 The transport of misfolded proteins and neuroinflammatory cytokines by EVs from different neuronal cells can increase the possibility of SCZ. Under such conditions, EV metabolites related to glycerophospholipids, phenylalanine, tyrosine, or tryptophan have been altered.^51^ Goetzl et al enriched astrocyte and neuron EVs using immunoprecipitation methods from the plasma of first-episode psychotics and controls. It was found that the levels of subunits 1 and 6 of complex I and subunit 10 of complex III were remarkably decreased, and ROS levels increased in the EVs of SCZ patients. The elevation of glial fibrillary acidic protein (GFAP) in EVs is closely associated with the polarization of astrocytes to the A1 type in SCZ patients. But this study has several limitations, including the challenge of precisely isolating and identifying EVs derived purely from neural sources in peripheral blood, considering the blood EV pool is a mixture of EVs derived from many tissues and cells that hampers capacity to identify changes that are uniquely related to brain pathology, and comparatively small sample sizes or heterogeneous patient populations may restrict statistical power and generalizability.^52^

 In another study, a noticeable difference was achieved in the plasma-derived EV lncRNAs, such as myocardial infarction-associated transcript (MIAT) and plasmacytoma variant translocation 1 (PVT1), between patients and controls.^53^ At varying degrees of activity, some types of miRNAs are essential in the pathophysiology of neuropsychiatric diseases. For instance, miR-132, which targets the CLOCK gene, is decreased in SCZ but increased in MDD and BD. miR-132 also has broad biological impacts, including the regulation of hippocampal neurogenesis, differentiation, axonal growth, neural plasticity, and the alleviation of memory deficits.^54^

 Like the alteration of genetic cargoes in EVs of SCZ, the lipid profile of plasma EVs can also be modified in SCZ candidates. Along with these changes, prefrontal cortex glycerophospholipid metabolism was decreased, and blood malondialdehyde (MDA) levels were elevated. These features indicate the role of lipid peroxidation in the pathogenesis of SCZ.^55^ The membrane phospholipid hypothesis of SCZ describes the critical role that phospholipids play in the development of the brain and its functioning. Based on this hypothesis, abnormal levels of essential fatty acids in the peripheral blood of SCZ individuals are integral to high-risk psychosis individuals, highlighting the focus on lipid metabolism as a therapeutic target for SCZ patients.^56^ Due to the current limited knowledge of the role of EVs in SZC, resulting from a lack of experimental results, further research is needed to identify the pathways involved in treatment and diagnosis.

Table 1 provides a summary of several relevant biomarkers on EVs, including proteins, nucleic acids, and lipids, described in neuropsychiatric disorders.

**Table 1:** Diagnostic importance of EVs in neuropsychiatric disorders

**Ref**	**Disease**	**Species**	**Biofluid**	**EV cargo**	**Diagnostic potential**
^57^	SCZ	Human	Plasma	higher ADE-P-T181-tau	Executive functioning in SCZ
^48^	SCZ	Human	serum	hsa-miR-206	Targets the BDNF mRNA
^48^	SCZ	Human	serum	hsa-miR145-5p, and hsa-miR-133a-3p	2-fold increase in SCZ
^58^	SCZ	Human	Astrocyte	Increasing miR-223	Targeting glutamate receptors
^59^	SCZ	Human	plasma	Increasing miR-137 and decreasing COX6A2	Cortical microcircuit impairment
^60^	SCZ	Human	Plasma	Higher GFAP and lower α-II-spectrin concentrations	Brain injury
^61^	SCZ	Human	blood	miR-137, miR-22-3p, miR-92A-3p	A combination of these factors can be used as biomarkers of SZ
^62^	Depression	Human	blood	Higher miR-186-5p and lower SERPINF1	Suppression of SERPINF1 in the hippocampus by miR-186-5p
^63^	Depression	Human	plasma	Insulin receptor substrate -1	increase in patients
^64^	Depression	Human	Serum	higher miR-9-5p	Increases microglia M1 polarization and exacerbation of depressive symptoms.
^65^	Depression	Human	plasma	miR-30d-5p, miR-486-5p, miR-21-5p	changed during antidepressant treatment and related to antidepressant treatment response
^66^	Depression	Human	blood	Higher IL-34	neuron-related blood biomarkers
^66^	Depression	Human	blood	Synaptophysin and TNFR1	positively associated with severities of depression
^67^	Depression	Human	plasma	Lower mitofusin 2 and cyclophilin D	neuron-related plasma biomarkers
^68^	Depression	Human	Serum	Upregulation of hsa-miR-139-5p	upregulated in patients
^69^	Depression and BD	Human	Blood	Higher miR-132	Target CLOCK gene
^70^	BD	Human	serum	sugar metabolism dysfunction	Molecular pathways underlying BP pathogenesis
^71^	BD	Human	blood	lower tryptophan, xanthurenic acid, kynurenine, and kynurenic acid	Have potential for use as biomarkers
^72^	BD	Human	plasma	p-JNK, p-p38-MAPK, p-ERK1/2	higher in BD patients treated with infliximab
^73^	BD	Human	serum	phosphatidylinositol	The level of phosphatidylinositol has an association with BD risk
^74^	Autism	Human	blood	Abnormal SVAT	A marker for the development of neural circuits
^75^	Autism	Mice	brain	Higher IL-1 and proinflammatory cytokines	Inflammation is a marker of autism
^76^	Epilepsy	Human	Plasma	hsa-miR-184 downregulation	Modulate apoptosis, inflammation, and neuronal death after an epileptic state
^77^	AD	Human	Plasma	miR-190-5p, miR-223-3p, miR-100-3p	showed a significant dysregulation
^78^	AD	Human	plasma	FGF, IGF-1, HGF	significantly lower in AD patients

Abbreviations: SCZ, schizophrenia; BD, bipolar disorder; AD, Alzheimer’s disease.

## Role of EVs in MDD

 Depression affects millions of people worldwide; it is one of the most prevalent mental disorders, and globally, the incidence is on the rise.^79^ In addition to a high recurrence rate and suicidal tendencies, patients also experience limited treatment effectiveness.^80^ Inflammatory hypotheses suggest that cytokines contribute to the pathophysiology of MDD and that changes in peripheral cytokine levels are associated with responses to antidepressants that include norepinephrine reuptake inhibitors (SNRIs), selective serotonin reuptake inhibitors (SSRIs), and serotonin.^81^ The etiology of MDD involves various mechanisms, including systemic immune activation, alterations in neurotransmitters and metabolism, dysfunction of the hypothalamic-pituitary-adrenal (HPA) axis, changes in the intestinal microbiome, and abnormalities in synaptic plasticity and mitochondrial function. Despite recent progress, no valid biomarkers have been detected to measure therapeutic response, disease state, or predict individual reactions to the therapeutic protocol.^82^ Due to the overlap between symptoms of MDD and other neuropsychiatric diseases, the diagnosis and prognosis of MDD require the detection of specific biomarkers.^83^ Studies targeting the role of EVs in MDD have provided important clues about potential mechanisms underlying this debilitating condition.^84^ Dysregulation of EV miRNA cargo has been observed among individuals with MDD, highlighting their involvement in regulating key molecular pathways relevant to depression.^85^ Furthermore, altered levels of EV-associated proteins involved in neuroplasticity and stress response have also been reported among MDD patients. The miRNA profile in neuron-derived plasma EVs of depressed patients showed the dysregulated miRNAs and their effect on postsynaptic density, cell growth, and axon formation, which can explain one of the reasons for a reduction in hippocampus volume in MDD patients.^86^ Microglia play a crucial role in MDD pathogenesis by inhibiting neurogenesis and the discharge of excitatory neurons through the secretion of EVs with higher levels of miR-146a-5p, which targets Krüppel-like factor 4 (KLF4). Additionally, EVs with higher miR-124 levels may enhance hippocampal neurogenesis and promote the polarization of M2 microglia.^87^

 To maintain CNS function and structure, astrocytes play a critical role, and their EVs contain miRNAs that regulate adult neurogenesis and stress responses.^88^ In normal conditions, astrocytes release EVs that mediate TGF-β signal activation through fibulin-2, resulting in synapse formation.^89^ EVs with miR-200a-3p have a neuroprotective role by downregulating MKK4, while the existence of fibroblast growth factor-2 (FGF-2) helps to maintain BBB integrity.^90^ The innate immune system, particularly aberrant Toll-like receptor (TLR) expression, may play a role in MDD etiology. During the development of MDD, negative regulators of TLR signal pathways control the magnitude and duration of cytokine storms induced by TLRs.^79^ It has been found that plasma EVs deliver sigma-1 receptors to the CNS, alleviate inflammation, and improve depression-like behavior in animal models.^91^ Fang et al showed that chronic stress affects serum EVs microRNA content, leading to alterations in important signaling pathways, such as MAPK, Wnt, and mTOR, and impairing neuroplasticity and inflammation. This led to changes in the levels of neurotrophic factors (i.e., BDNF) and synaptic proteins in mood-regulating brain regions (e.g., the hippocampus and prefrontal cortex, or PFC). Altered microRNAs in EVs also disrupt neurogenesis and synaptic functions in these regions, providing insight into the depressive-like behaviors observed. Furthermore, these findings could have implications for EVs’ microRNAs as biomarkers for early detection and potential therapeutic targets for stress-induced depression.^92^ However, the study has limitations that should be noted, including that the study reported protein expression levels of important markers (BDNF, TrkB, synaptotagmin 1) only in the hippocampus and prefrontal cortex, and did not report depression-related changes in any other brain regions that may be important to consider. The study used a chronic unpredictable mild stress model in rats, and while a widely recognized behavioral model of depression, it may not reflect the entirety of human depression pathophysiology. Hung et al found the reduction of only miR-146a in serum EVs of MDD patients before antidepressant medication, and the levels of let-7e and miR-155 were increased following treatment with antidepressants. Compared to the remission group, the non-remission group exhibited significantly higher levels of let-7e, miR-21-5p, miR-223, and miR-146a, indicating that EVs negatively affect TLR4 signaling through the regulation of these microRNAs.^79^ The limitations of this study are the following: Variation in patient characteristics, severity of illness, medication regimens, and co-morbid conditions may bias miRNA expression and biomarker validity, and divergence in EV isolation methods may bias results by variability in miRNA extraction, extraction methods, quality control, and quantification. Wei et al found that hsa-miR-139-5p was significantly higher in MDD patients than in the control group. They isolated exosomes from the blood of MDD patients and injected them into normal mice. The results indicated that this group of mice showed depressive-like behaviors and had remarkably fewer DCX + cells in the subgranular zone, a marker of neurogenesis. Also, they isolated blood exosomes from healthy volunteers and injected them into mice models of depression that were subjected to chronic unpredictable mild stress. Compared to the control, this group of mice had a notably decreased total antioxidant capacity (T-AOC) and increased MDA levels, which are two markers for assessing oxidative stress.^93^ While the study identified EVs induced changes in oxidative stress and neurogenesis markers, neither the specific mechanisms nor the degree to which blood EVs enter the BBB to alter brain function could be determined.

 It has been proposed that astrocytes are implicated in the pathophysiology of depression and could serve as a significant therapeutic target.^94^ These cells are the prime source of inflammatory cytokines.^95^ As a component of the TLR4 signaling pathway, leucine-rich repeats (Tril) are highly expressed in astrocytes and mediate pro-inflammatory changes by activating transcription factors.^79^ NK cell EV miR-207 may increase the release of monoamine factors in the brain by targeting Tril, thereby improving the behavior of mice and reducing the secretion of inflammatory factors.^96^ Ran et al conducted miRNA sequencing of serum EVs and identified 18 upregulated and 14 downregulated miRNAs in adolescents with childhood MDD. Of them, miR-450a-2-3p, miR-556-3p, and miR-2115-3p were remarkably different, especially miR-450a-2-3p. They also indicated the link between miR-450a-2-3p and trauma in childhood and MDD in adolescents.^22^ However, the study’s limitations include a relatively small sample size, which limits the generalizability and robustness of the findings. Additionally, the study did not examine the mechanistic underpinnings by which dysregulated miRNAs affect the pathophysiology of depression.

 Significantly higher levels of microglial GLS1 mRNA, along with the neuroinflammation pathway, were reported by Ji et al in the prefrontal cortex of the depression model. Reduced neuroinflammation by GLS1-deficient microglia was a consequence of less reactive astrocytes, as GLS1 deficiency increases miR-7115-3p and miR-666-3p levels in EVs released from microglia, therefore suppressing astrocyte activation via blocking Serpina3n expression. These results highlight the promise that EVs hold as biomarkers that aid in comprehension, diagnosis, and the development of more effective treatments for MDD.^97^ A recent study elevated miR-182-5p levels as a biomarker and modulator of MDD, and the dysregulated miR-182-5p in EVs, known to play a role in depression through regulating critical genes related to HPA activity (e.g., FKBP5 and CRHR2), which control stress reactivity, will allow further examination of the EV-derived miR-182-5p in the potential for depression’s pathophysiology. The functional experiment of injecting MDD patient-derived EVs into the paraventricular nucleus (PVN) of mice demonstrated miR-182-5p-related HPA axis hyperactivity, as indicated by increased expression of corticotropin-releasing factor (CRF). Bioinformatics analysis also indicated pathways related to miR-182-5p, including protein transport, cytoskeletal regulation, and glutamatergic synapses, which relate to a depressive pathology. miR-182-5p within plasma sEVs contributes to MDD by modulating HPA axis dysfunction and synaptic changes, demonstrating the potential for vesicular miRNAs as biomarkers for diagnosis, treatment, and prevention of depression.^98^

 In a recent paper, researchers reported that EVs derived from human umbilical cord mesenchymal stem cells (hUCMSCs) exhibit antidepressant effects by targeting specific pathophysiological pathways associated with depression. They found that these EVs produce a significant reduction in neuroinflammation by inhibiting pro-inflammatory cytokines, such as IL-1β and TNF-α, thereby normalizing the morphology of microglial activation in the hippocampus. Furthermore, as indicated by increased expression of BDNF, they promote neuronal survival and synaptic plasticity. EVs were also reported to stimulate neurogenesis and enhance the dendritic complexity of doublecortin-positive cells in the dentate gyrus, thereby restoring vulnerable interneuron connections after chronic stress. Moreover, their small size also promotes biodistribution and the ability to cross the blood-brain barrier, supporting immunomodulation in systemic circulation and the CNS. These various modulations of neuroimmune and neurotrophic systems by hucMSC-EVs contribute to the alleviation of depressive-like behaviors, making them a novel and efficacious intervention for treating depression.^99^

## Role of EVs in AD

**Figure 3:**
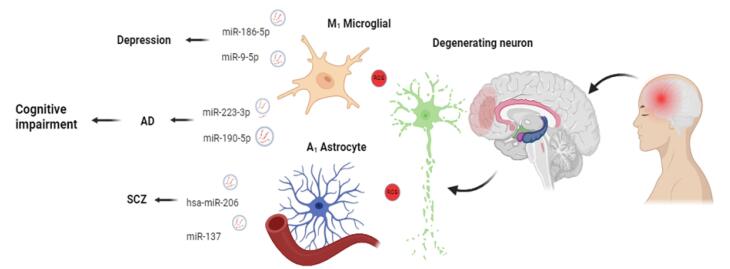
The elevation of EV miR-186-5p is common in MDD. These features contribute to the suppression of SERPINF1 in the hippocampus. Besides, the increase of EV miR-9-5p promotes the polarization of microglia toward the M1 phenotype, resulting in the exacerbation of depressive symptoms. AD is characterized by significant dysregulation of EV miR-190-5p and miR-223-3p. Similar to AD and MDD, the levels of hsa-miR-206 (targeting BDNF mRNA), and miR-137 in EVs increase in the blood EV of SCZ patients. The alteration of genetic elements and various cargoes contributes to cognitive impairment and neuropsychiatric diseases

 The ageing of the global population is leading to an annual increase in the number of AD patients and making it a major public health concern.^100^ A person with AD is most likely to be diagnosed once irreversible neuronal damage has occurred, as it is one of the conditions among the principal mortality causes without a preventative treatment or cure.^101^ AD symptoms mainly include neuropsychiatric abnormalities, progressive memory decline, and cognitive impairment.^102^ In AD pathogenesis, there is a silent accumulation of proteins, and symptoms do not occur until late pathological conditions.^103^ Individuals’ cognitive performance declines after this silent period, and memory and executive cognitive alterations first appear. The first molecular changes in AD pathogenesis can occur up to 15 years before symptoms appear.^104^ The most extensive study of EVs in the brain has been conducted on AD to date. The pathology of AD is confirmed by the presence of amyloid beta (Aβ) plaques and the formation of hyperphosphorylated tau (p-tau) tangles.^105^ The tau protein exists in several forms, including p-T181-tau, and p-S396-tau, and these molecules have been found in neuron EVs of AD patients compared to healthy controls.^106^ CSF-tau and CSF-Aβ can be used as diagnostic biomarkers for monitoring AD status.^107^ Studies have shown that with AD progression, the levels of EV Aβ42 protein are also increased.^108^ Other factors like growth-associated protein 43 (GAP43), neurogranin (NRGN), synaptosome-associated protein 25 (SNAP25), synaptotagmin, and repressor element 1-silencing transcription factor (REST) are statistically reduced.^109^ In a study by Kumar et al, the miRNA level and profile were found to be significantly different in plasma EVs of adults with mild cognitive impairment (MCI) and AD as compared to healthy individuals. They suggest downregulating miR-106b and miR-29a-5p, and overexpressing miR-106b-5p, miR-132-5p, and miR-106b-5p.^110^ The key limitations of the study are a moderate sample size of the population. Notably, this study cannot exclude potential confounders from peripheral sources of EVs, as blood EVs can originate from multiple organs and have been reported to differentiate in response to trauma or health states unrelated to brain pathology. In neuron-derived plasma EVs, the levels of miR-223-3p, miR-23a-3p, miR-190-5p, and miR-100-3p are between AD patients and controls (Figure 3). Along with these changes, the levels of FGF-2 and −13, hepatocyte growth factor (HGF), and type 1 insulin-like growth factor (IGF-1) were lowered in AD patients.^111^ Neurexin 2α, GluA4-containing glutamate receptor, neurogranin, and neuronal pentraxin 2 are proteins that originated from neuronal cells and are significantly decreased in the neuronal-derived plasma EVs of AD patients.^112^ According to a study by Koinuma et al, EV secretion affects both intracellular and extracellular Aβ levels. It also downregulates autophagosomes through siRNA. These findings imply that EVs are an important source of Aβ in the brain and that abnormal Aβ accumulation in EVs may play a role in the transition of age-dependent Aβ pathology.^113^

 Chen et al showed that cognitive deficits of AD, degradation of plaques, and brain glucose metabolism can be significantly improved by using mesenchymal stem cell (MSC)-derived EVs.^114^ Li et al found that bone marrow MSC (BMSC) EVs alleviated AD-like behaviors in a mouse model by increasing the expression of synapse-related proteins and BDNF, reducing the levels of IL-1β, IL-6, and TNF-α, and suppressing the hyperactivation of microglia and astrocytes.^115^ The limitations of this study are primarily related to the use of an AD animal model, which may not fully encompass all aspects of human AD pathology, thereby limiting clinical translation. The molecular mechanisms underlying the neuroprotective effects of BMSC-derived EVs, including their impact on BDNF pathways and neuroinflammation, require further exploration. High-throughput sequencing of EVs from hypoxia-pretreated adipose-derived stem cells (ADSCs) showed improved cognitive function. Based on the data, circ-Epc1 was an essential component of hypoxia-pretreated ADSC EVs with the potential to enhance mental function and microglia polarization from M1 to M2 type in AD mice.^116^ Li et al demonstrated that M2 microglia-derived EVs, when internalized by neurons, exhibited neuroprotective functions, increased cell viability, decreased Aβ plaque deposition, and improved the PINK1/Parkin pathway of autophagy.^117^ Iyaswamy et al presented engineering EVs that overexpress Fe65 and carried Corynoxine-B as an autophagy inducer. These EVs hijacked the interaction between Fe65 and APP, induced autophagy in amyloid precursor protein (APP)-expressing cells, and ameliorated the cognitive decline and pathogenesis in the AD model.^118^ The limitations of this study include the need to thoroughly assess the long-term safety of engineered EVs and any potential immune response triggered by their in vivo introduction into humans. Although the targeted delivery of engineered EV was beneficial, assessing off-target effects and biodistribution to other organs outside the brain, such as the liver and spleen, should always be conducted.

 The application of EVs in clinical practice is still in the early stages, but advances in engineered exosomes for targeted drug delivery and purification technology could allow these findings to be applied.

 The brain is comprised of approximately 86 billion neurons interconnected by trillions of synapses.^119^ This intricate communication network forms the basis of cognitive functions, emotions, and behavior. When an electrical impulse reaches the end of a neuron, it triggers the release of neurotransmitters into the synaptic space. These neurotransmitters bind to receptors on the adjacent neurons, generating a new electrical signal and continuing the transmission of information.^120^ In recent years, the increasing importance of exosomes in modulating synaptic communication has added a new dimension to our understanding of neuropsychiatric disorders.^121^ Aberrant exosome-mediated communication maycontribute to the synaptic dysfunction observed in conditions like MDD.^122^ In certain areas of the cerebral cortex and the hippocampus, interactions between the presynaptic proteins neurexin 2α (NRXN2α) and neuronal pentraxin 2 (NPTX2), along with their respective postsynaptic functional partners, the GluA4-containing glutamate receptor (AMPA4) and neuroligin 1 (NLGN1), enhance excitatory synaptic activity. Since the cognitive decline in AD participants is associated with early damage to these excitatory circuits in their brain tissues, levels of all proteins in plasma neuron-derived EVs, except for NPTX2, were significantly lower in the years before the onset of dementia, and levels of all proteins decreased significantly as dementia progressed.^123^ Moreover, the research by Cui et al provides evidence that EVs obtained from hypoxia-preconditioned mesenchymal stromal cells (MSCs) are capable of preventing memory deficits in AD by rescuing synaptic dysfunction and regulating inflammatory responses.^124^

## Role of EVs in autism spectrum disorder

 Autistic spectrum disorder (ASD) is a neurodevelopmental disorder, and it has been estimated that nearly 200,000 cases of autism occur per year.^125^ A large number of autistic patients suffer from intellectual disability and have lifelong developmental disabilities in adulthood due to severe blockages in neurological and mental development.^126^ Based on the statistics, ASD has a substantial economic and psychological impact on society and families.^127^ The symptoms of autism can generally be categorized into two main areas: social communication and repetitive behaviors. Children with autism may struggle to make eye contact, have difficulty understanding nonverbal cues, and have trouble initiating or maintaining conversations.^128^ There is no cure for autism, but early intervention and therapy can help improve outcomes for ASD children. Most therapies, for instance, special education, adjuvant drug therapy, and targeting intestinal microbes, cannot effectively improve the symptoms of autism patients.^129^ As SVAT-related RNA expression changes were detected in blood EVs of ASD patients, monitoring SVAT changes could be used to predict neural circuit development in the fetal brain.^74^ Researchers have found that EVs in the serum of ASD patients cause microglia to have more proinflammatory cytokines, particularly IL-1. In comparison with other sources of EVs, it is believed that umbilical cord MSCs can secrete EVs containing immune-suppressing factors, such as IL-10 and TGF-β, to inhibit pathological immune responses.^28^ MSC-EVs could alleviate repetitive behavioral phenotypes and have potency as cell-free, noninvasive therapies. They are also better clinical options due to their advantages in immunogenicity, clinical safety, ethics, and stability, as well as their simplicity in transportation and storage. A long-term improvement in the behavioral phenotype of autism was achieved by transplanting human bone marrow stem cells (BMSCs) into the lateral ventricles of BTBR mice, where EVs were the primary therapeutic mediators.^75^ The intranasal administration of human MSC EVs in rats with experimental perinatal brain damage reduced microglia-mediated neuroinflammation. This suggests that neuroinflammation, increased pro-inflammatory cytokine levels, and abnormal microglia/astrocyte responses play an important role in autism pathogenesis in autistic brains and animal models of autism.^130^

 The results from a study by Qin et al show that engineered EVs bearing miR-137, which are modified with rabies virus glycoprotein (RVG) for targeting, ameliorate autism-like behaviors through neuroinflammation modulation and effects on brain glucose metabolism. MiR-137 could be increased in the brain after RVG-miR137-EVs treatment (the levels of miR-137 are decreased in autistic model mice and human patients), which lowers the activation of microglia and pro-inflammatory cytokine levels IL-1β and TNF-α. Mechanistically, the miR-137 targets and downregulates Toll-like receptor 4 (TLR4), thus inhibiting the TLR4/NF-κB signaling pathway. The downregulation of TLR4 decreases hypoxia-inducible factor 1α (HIF-1α) expression along with the essential glycolytic enzymes HK2, PKM2, and LDHA, resulting in decreased glycolysis and lactate function associated with the Warburg effect in activated microglia. Pharmacological inhibition of TLR4 or glycolysis, acting in parallel, decreases neuroinflammation and autism-like behaviors. In summary, miR-137–mediated repression of the TLR4/NF-κB/HIF-1α axis restores microglial metabolic and inflammatory behaviors, leading to improved neurological outcomes.^131^ This study needs further investigation of long-term effects, as behavioral tests were conducted at a single time point and may not fully capture the persistent therapeutic effects. The specific molecular mechanisms by which miR-137 modifies glucose metabolism and neuroinflammation through the TLR4/NF-κB and HIF-1α pathways also require further elucidation.

Table 2 provides a summary of EVs used for the treatment of various neuropsychiatric disorders.

**Table 2:** Therapeutic Potential of EVs in Neuropsychiatric Disorders.

**Reference**	**Diseases**	**Species**	**Source of EVs**	**Contents of EVs**	**Outcome**
^132^	Depression	Mice	NK cells	miR-207 overexpression	Reduced inflammation and improved depressive symptoms
^133^	Depression	rats	BMSC)-derived exosomes	upregulating miR-26a	Alleviate neuronal injury in the hippocampus
^134^	Autism	Mice	umbilical cord-derived MSC	IL-I0 and TGF-β	Ameliorated symptoms of Autism
^114^	AD	Transgenic mice	Wharton’s jelly MSCs	HDAC4-targeting miRNA-29a	Improve cognitive deficits and brain glucose metabolism
^116^	AD	Mice	ADSCs	circ-Epc1	shifted microglial M1/M2 polarization, and improved cognition
^117^	AD	Mice	M2 Microglia	miR-124-3p	neuroprotective functions and improved PINK1/Parkin pathway of autophagy.

## Role of EVs in posttraumatic stress disorder (PTSD)

 PTSD is one of the neuropsychiatric disorders that develops after experiencing a traumatic event, and its symptoms include intrusive thoughts, flashbacks, avoidance behaviors, and hyperarousal. Research indicated alterations in brain structure and function of patients with PTSD, such as a reduction in the volume of the hippocampus and abnormalities in the amygdala.^135,136^

 The research on EVs in PTSD is still in its nascent stages, with a significant scarcity of large-scale studies compared to disorders like MDD or AD. Therefore, the current evidence is preliminary but points to several intriguing mechanisms. Kang et al demonstrated that changes in the expression of a set of circulating miRNAs associated with EVs, specifically related to the FK506-binding protein 5 (FKBP5) gene, are linked to PTSD.^137^ Lee et al also analyzed miRNAs in blood derived from PTSD patients and controlled by NGS and observed an increase in the concentration of miR-203a-3p. miR-203a-3p is associated with inflammatory pathways, serotonergic signaling, and neurotransmitter systems: various signaling pathways, cell cycle, inflammation, and metabolism.^138^ In a study, Bam et al found that miR-7113-5p is decreased in Peripheral Blood Mononuclear Cells (PBMCs) of PTSD patients. The researchers also showed that this downregulation of miR-7113-5p promotes an inflammatory response by targeting and upregulating the WNT signaling pathway.^139^ However, the sample size was small, and it is essential to replicate the study with larger and more diverse populations. Additionally, the role of dysregulated Wnt signaling as a driver of inflammation is supported; however, because the modulation of the Wnt pathway is context-dependent, we do not yet fully understand the downstream effects on the various immune cell subsets, nor the brain-immune interactions. Guo et al showed that EVs from PTSD subjects transport the chemokine CXCL8 through the protein CD63, which strengthens communication between astrocytes and neurons. These EVs exacerbated anxiety- and depression- like behavior in a PTSD mouse model, and even though silencing CXCL8, improved these symptoms. The authors conclude that the CD63-mediated delivery of CXCL8 via EVs is an important mechanism driving PTSD and a potential therapeutic target.^140^

## Role of EVs in epilepsy

 Epilepsy is characterized by abnormal discharges of neurons mostly in the hippocampus, amygdala, temporal, and frontal lobes,^141^ and the underlying reason for its onset is genetics, brain injuries (such as a stroke or a traumatic brain injury), developmental disorders, infections, and tumors. Diagnosis of this disorder is performed by evaluating the patient’s medical history, conducting a physical examination, performing electroencephalography (EEG), and utilizing brain imaging.^142^ In a study that characterized the effects of experimental status epilepticus on mouse hippocampal EVs and their miRNA content, miR-21-5p exhibited strong changes in CSF samples. miR-21-5p has been shown to regulate neurotrophin 3 mRNA and Mef2c transcripts, which encode neural transcription factors in the hippocampus. The efficacy of miR-21 in EVs may be associated with the status epilepticus or epileptogenesis. It has been shown that it may be useful in diagnosis.^143^ miR-346 and miR-331-3p were significantly downregulated in EVS from anterior brain cells in an animal model of epilepsy.^144^ Additionally, miR-21a-3p and miR-21a-5p, as well as miR-146a-5p, have been shown to be upregulated in animal models and humans.^143^ In addition to miR-194-2 and miR-15a, serum EVs in epilepsy patients were downregulated, and higher coagulation factor IX (F9) and lower thrombospondin-1 (TSP-1) were detected.^145^

 Yu et al demonstrated that miR-106b-5p level increased and repulsive guidance molecule A (RGMa) and triggering receptor expressed on myeloid cell 2 (TREM2) levels decreased in the lithium-pilocarpine-induced Epilepsy mouse model. This finding indicated inflammation and microglial activation in the hippocampus of the Epilepsy mouse model.^146^ Mesenchymal stem cell EVs have been shown to have anti-inflammatory properties and restore activation of hippocampal astrocytes.^147^

 Ballal et al demonstrated that exogenous GABA can be used to alleviate seizures in epilepsy when the GABA is carried and delivered by EVs derived from certain brain cells. EVs from GABAergic interneurons and EVs from medial ganglionic eminence cells reduced seizure frequency and severity in a rat model of epilepsy. Also, they improved depressive behavior and some memory functions. Cell-specific EV-mediated delivery of GABA provides a potentially exciting new treatment modality for the management of epilepsy.^148^ Nevertheless, these observations are limited due to rats with kainic acid-induced epilepsy being used in this study, and do not wholly represent the diversity, complexity, and multiple variants of human epilepsy. This study did not provide a rigorous evaluation of potential long-term effects and off-target effects with repeated dosing of EVs.

## Role of EVs in bipolar disorder

 BD is characterized by extreme mood changes that alternate between episodes of mania and depression, and Genetics and the environment both play a role in the development of this disease. During manic episodes, the symptoms include excessive energy, low need for sleep, and impulsivity. In contrast, during depressive episodes, patients experience sadness, hopelessness, low energy, and difficulty concentrating.^149^ The delay in diagnosis may lead to neuroprogression, which could be due to a cumulative effect of immune dysfunction, loss of neurotrophic factors, and increased oxidative stress.^122^ Research findings link EVs to the development and progression of BDs. Neuronal excitability in BP could be influenced by the EV-mediated transfer of miRNAs between neurons, which is dysregulated in patients.^150^

**Figure 4:**
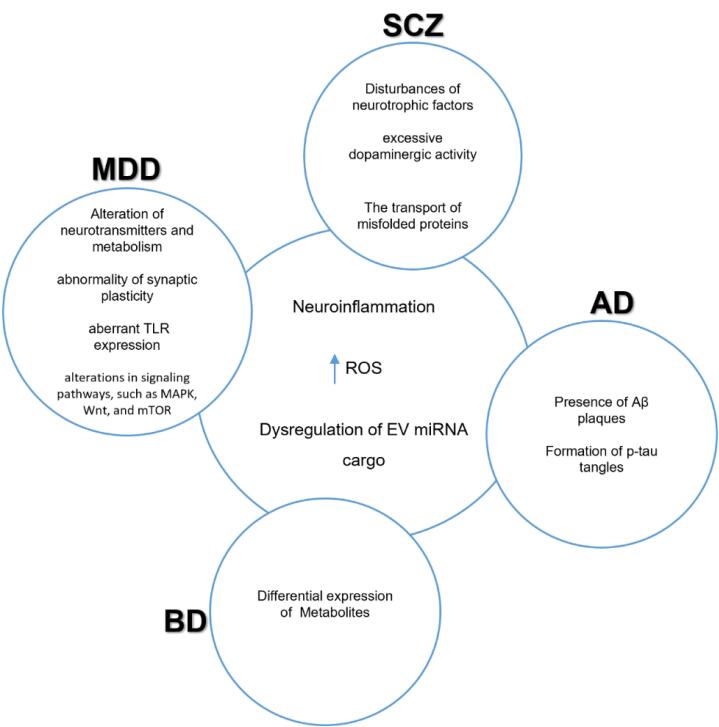
Shared pathological mechanisms in neuropsychiatric disorders. schizophrenia (SCZ), major depressive disorder (MDD), Alzheimer’s disease (AD), and bipolar disorder (BD) have shared pathological mechanisms that include neuroinflammation, increased reactive oxygen species (ROS), and dysregulation of EV cargo composition^151^

 Ceylan et al analyzed the expression of miRNA in EVs of BD patients. The observation indicated significant downregulation of MiR-484, -652-3p, -142-3p, and remarkable upregulation of miR-185-5p. They performed bioinformatic analysis and identified several target pathways, including the PI3K/Akt signaling pathway, adhesion pathways, fatty acid biosynthesis/metabolism, and the extracellular matrix.^151^ Du et al performed ultraperformance liquid chromatography-tandem mass spectrometry (UPLC-MS-MS) and examined the serum-derived EVs of BD patients. They presented a total of 26 EV metabolites that exhibited differential expression in patients diagnosed with BD (n = 32) when compared to the control group. Furthermore, these differentially expressed metabolites were found to be significantly enriched in pathways associated with sugar metabolism.^70^ Study limitations include the use of a small sample size (32 BD patients and 40 healthy controls). Some confounders were not measured, such as smoking status, socioeconomic status, and the severity of the disease, which could affect the levels of the measured metabolites (Figure 4). The cross-sectional nature of this study does not permit firm conclusions about the changes in metabolites as the disease progresses.

 Fries et al analyzed EV miRNAs in the blood of BD patients and controls, and their results revealed notable differences between the two groups of miRNAs, including miR-29c and miR-22. These miRNAs are involved in various signaling pathways in the brain, including serotonergic receptor type 5HT2 and netrin-mediated signaling pathway.^152^

## Clinical translation

 The clinical translation of engineered EVs presents numerous challenges, particularly in relation to regulatory approvals, reproducible manufacturing processes, and the design of well-structured clinical trials. A primary challenge concerning regulatory permissions involves the classification of EVs as drug products rather than as diagnostics. The critical regulatory issue pertains to establishing drug status, as the Food and Drug Administration (FDA) categorizes all EV-based therapeutics as biologics, which fall under the jurisdiction of its Center for Biologics Evaluation and Research. These products are required to adhere to the FDA’s stringent Investigational New Drug (IND) application process, necessitating comprehensive documentation about safety, efficacy, and purity. This process includes substantiating EV targeting and clearance through detailed biodistribution studies associated with the use of EVs. Moreover, it is imperative to document the immunogenicity and toxicity of the EVs, ensuring compliance with the minimum safety threshold established for regulated products. Given the biological heterogeneity of EV populations and their constituent materials, standardizing protocols for the production of engineered EVs in accordance with Good Manufacturing Practice (GMP) remains a significant challenge, particularly when compared to conventional therapeutic products. In addition to the complexities related to nanoparticles, efforts must be directed toward minimizing batch variability, scale-up manufacturing of EVs from bacterial, mammalian, or plant cell sources without compromising their integrity or potency, ensuring that all preparations are free from contaminants, and developing validated potency assays to confirm the therapeutic efficacy of the EVs. Furthermore, the selection of endpoints for clinical trials must be carefully tailored to address disease-specific requirements. This includes the analysis of EV biomarker levels, which may necessitate the incorporation of validated cognitive assessments for neuropsychiatric disorders such as MDD or BD. It is also essential to consider the impact of functional imaging techniques (e.g., fMRI or PET) on multi-modal assessments, which aim to encompass both the overall clinical response of symptoms and the biological mechanisms at play.^153,154^

## Conclusion

 The analysis of EVs in neuropsychiatric disorders enhances our understanding of their roles in gene expression and intercellular communication. This research underscores the potential of EVs as therapeutic targets and precise diagnostic tools. However, these studies have not yet transitioned into clinical applications due to several limitations, including the limited availability of disease-specific EV-based biomarkers, ambiguity concerning the precise functions of EVs, inconsistent research outcomes, small sample sizes, a lack of standardized detection methodologies, and concerns regarding the reliability of EVs. Recent investigations using proton magnetic resonance spectroscopy and high-throughput sequencing have aimed to identify potential biomarkers that can distinguish between various neuropsychiatric conditions. In contrast to invasive procedures such as tissue biopsies and lumbar punctures, EVs present numerous advantages in clinical practice, including the ability to reflect brain pathophysiology without requiring surgical interventions, thereby enhancing prognosis and facilitating the monitoring of disease progression. Consequently, there is a need for the development of methods based on the hallmarks of EVs associated with the early stages of neuropsychiatric disorders, which would allow for non-invasive, cost-effective, population-wide screening. Despite the lack of clear pathological features in neuropsychiatric diseases, omics exploration has revealed new potential protein and miRNA biomarkers that may assist in predicting disease status. Ongoing research in this domain has the potential to elucidate the underlying mechanisms of these disorders and contribute to the development of effective diagnostic and therapeutic tools. The capacity of EVs to deliver disease-specific biomarkers while providing therapeutic cargo signifies their promise as combined theragnostic platforms. Future studies must focus not only on the independent development of diagnostics and therapies but also on integrating both elements to create personalized diagnostic and treatment approaches for neuropsychiatric conditions. This review aims to summarize the advancements in the application of EVs as biomarkers and treatment modalities for neuropsychiatric disorders, which carry significant implications for clinical utilization.

## Competing Interests

 The authors declare that they have no conflicts of interest.

## Ethical Approval

 Not applicable.
